# Neck- and balance related complaints in subjects with visual impairment and ongoing inpatient-rehabilitation: an exploratory cross-sectional study

**DOI:** 10.1038/s41598-026-58352-x

**Published:** 2026-06-16

**Authors:** Bernd Brechtelsbauer, Oliver Kolbe, Dino Capovilla, Kathleen S. Kunert

**Affiliations:** 1 Institute of Rehabilitation Medicine, Medical Faculty, Martin Luther University Halle-Wittenberg, Magdeburger Straße 8, 06112 Halle (Saale), Germany; 2 Henneberg Rehabilitation Clinic for Ophthalmology, Hauptstraße 18, 98666 Masserberg, Germany; 3 Chair of Pedagogy for Visual Impairments and General, Special and Inclusive Education, Julius-Maximilians-University of Würzburg, Wittelsbacherplatz 1, 97074 Würzburg, Germany

**Keywords:** Vision-related Quality of life, Visual impairment, Musculoskeletal complaints, Neck pain, Balance, Diseases, Health care, Medical research, Signs and symptoms

## Abstract

Neck pain, impaired cervical motor control, fear of falling, and balance limitations may be relevant but under-recognized concerns in inpatient ophthalmologic rehabilitation for patients with visual impairment (VI). To investigate whether onset/duration and type of VI influence neck and balance complaints in visually impaired patients. Exploratory cross-sectional study of 80 participants (median age 58 years; 43 women, 37 men). Outcomes included neck pain (NRS), neck disability (NDI), cervical motor control, fear of falling (FES-I), balance (FABS), and vision-related quality of life (NEI VFQ-25 C). Associations were analyzed by VI duration (Group I < 1 year; Group II > 3 years; Group III 1–3 years) and VI type (unilateral; unilateral blindness; bilateral; bilateral blindness; other; not classifiable). Neck pain was reported by 71.3% (NRSmean 4.6/10, SD 1.7 (median: 4.0 (IQR: 2.0–7.0)); NDI 13.5/50, SD 6.0). Participants performed a mean of 2.3/4 motor control tests incorrectly (SD 1.1). Fear of falling was moderate (FES-I 24.8/64, SD 7.7), balance performance was below age average (FABS 33.2/40, SD 3.6), and NEI VFQ-25 C averaged 0.65 logits (SD 1.91). Higher fear of falling was associated with longer-lasting VI (f = 0.31; χ² = 8.94; *p* = .01) and with more severe VI types (f = 0.41; χ² = 10.82; *p* = .03). This assessment captures frequent neck pain, impaired cervical motor control, increased Fear of Falling and non-age appropriate Balance in this population. Findings show greater Fear of Falling with longer lasting VI-Symptoms and increasing VI severity, ranging from visual impairment to blindness and from unilateral to bilateral involvement. *Trial registration *German Clinical Trials Registration-Number: DRKS00035125, registered on 30.09.2024.

## Introduction

Visual impairment (VI) and blindness are associated with substantial psychological distress, high disability rates, and various comorbid conditions. According to the German Federal Statistical Office, 328,680 individuals were registered in the 2023 severe disability statistics under the category “blindness and visual impairment” (63,425 blind, 41,245 severely visually impaired, and 224,015 visually impaired persons)^1^. However, the actual number of people suffering from a visual impairment is likely to be much higher, as the statistics only record those who have been classified as severely disabled (degree of disability ≥ 50%). Unilateral and less severe visual impairments are not included in the statistics, even though they can already result in significant limitations in activities of daily living and social participation for those affected.

Despite the high prevalence and impact of visual impairment, the musculoskeletal dimension in this patient population has received insufficient scientific attention. The literature points to an increased incidence of shoulder and neck complaints in patients with age-related macular degeneration^2^, as well as associations between visual strain and the development of neck pain^3^. Ocular symptoms such as redness, dryness, itching, or grittiness are also linked to neck pain^4^. Moreover, there is an interaction between visual strain and the EMG activity of the descending part of the trapezius muscle, whereby deviations in refractive lighting conditions increase muscular activity^5^.

Psychosocial factors may further contribute to the clinical relevance and persistence of cervical complaints in this population, such as stress^6^, anxiety^7^, depression^8^, sleep disturbances^6^, loneliness^9^, lack of social support^10^, and unhealthy dietary habits^11^. Even mild visual impairments threaten social position and frequently lead to unemployment (risk ratio 1.55) or employment in low-skilled or poorly paid occupations (risk ratio 1.24)^12^.

The underlying mechanisms remain unclear. Forced head postures associated with visual impairment are observed in only 18–25% of cases^13^. Consequently, potential causes for the increased prevalence of neck pain among individuals with visual impairments include not only mechanical factors but also cervicocephalic or oculocervical reflexes, psychosocial stressors, or a combination thereof.

In addition to neck complaints, both children^14^ and adults^15^ with visual impairments demonstrate reduced balance ability compared to sighted individuals. They are more frequently affected by falls^16^, which more often result in serious consequences such as hip fractures^17^.

Based on these findings, the primary objective of the present study is to investigate the relevance of cervical spine complaints and balance related impairments in individuals with visual impairment, considering both the duration and type of visual impairment. Secondary objective is to explore associations between Neck Pain and Disability, Balance, Fear of Falling and vision related Quality of Live.

## Methods

The present study was designed as an exploratory, monocentric cross-sectional study and was conducted at the Henneberg Rehabilitation Clinic for Ophthalmology (Masserberg, Germany) in May 2023. All participants provided written informed consent; the study complied with the Declaration of Helsinki and was approved by the Ethics Committee of the University of Applied Sciences Osnabrück in April 2023 (Germany; wiso_MS-MT _HP-SS-23-02). Registration in the German Clinical Trials Register took place retrospectively due to publication process.

### In- & exclusion criterias

Inclusion criteria, besides inpatient treatment with a primary ophthalmological referral diagnosis, are only age over 18 and willingness to participate. Exclusion criteria include a history of cervical spine surgery, systemic diseases (multiple sclerosis, amyotrophic lateral sclerosis, rheumatoid arthritis, ankylosing spondylitis, etc.), vertigo, tinnitus, temporomandibular joint dysfunction, combined visual and hearing impairments, and insufficient German language skills.

### Measurement instruments

To assess the intensity of neck pain and functional limitations of the cervical spine, the Numeric Rating Scale (NRS; 0 = no pain, 10 = maximum pain) and the Neck Disability Index (NDI; 0–4 = no disability, 5–14 = mild, 15–24 = moderate, 25–34 = severe, 35–50 = complete disability) were used^18^. Fear of falling was measured using the Falls Efficacy Scale International (FES-I; 16–19 = no concern about falling, 20–27 = moderate concern, 28–64 = high concern)^19^.

Balance ability was quantified using the Fullerton Advanced Balance Scale (FABS; 0 = poorest score, 40 = best score, < 22 = increased fall risk)^20^. Motor control was assessed using a brief screening consisting of four cervical motor tasks as described by Luomajoki et al.^21^: transverse head protraction and retraction (Inter-tester reliability K = 0.91), deep cervical extension (K = 0.68), isolated rotation in quadruped position (K = 0.47)^22^, and an eye-head coordination task (weighted K = 0.72)^23^. The execution of the movement was rated by the assessor on a binary scale as either correct or incorrect. Vision-related quality of life was assessed using validate German version of the National Eye Institute Visual Function Questionnaire NEI VFQ^24^, raw data was Rasch-calibrated to the NEI VFQ-25 C according to Goldstein et al.^25^. MCID thresholds were additionally calculated as described in the statistical analysis.

The examiner was unaware of the duration and type of subjects visual impairment. Nevertheless, some of the visual impairments are obvious and, as such, interpretable, hence masking might be compromised.

### Group classification

To investigate the multidimensional impact of visual impairment on musculoskeletal and balance-related outcomes, participants were classified according to two clinically relevant dimensions: duration of visual impairment and type and laterality of impairment.

Participants were grouped by the duration of visual impairment (Group 1: <1 year; Group 2: > 3 years; Group 3: between 1 and 3 Years) to examine potential adaptation and chronification processes. This temporal classification is grounded in the understanding that musculoskeletal complaints and balance impairments may evolve over time as individuals develop compensatory strategies or, conversely, experience progressive deconditioning. Recent visual impairment may be associated with acute postural adaptations and increased fall risk due to lack of compensatory mechanisms, while longer duration may reflect either successful adaptation or chronic musculoskeletal overload from sustained compensatory postures. Additionally, the duration of impairment may influence psychosocial factors such as depression and social isolation, which are known risk factors for chronic pain.

A deviation from the standard WHO classification regarding the severity of visual disability was necessary because the rehabilitation population could not be consistently categorized within it. The WHO classification focuses predominantly on bilateral impairments based on the better-seeing eye, thereby systematically excluding individuals with unilateral visual impairment or blindness, despite evidence that even monocular impairment causes significant limitations in activities of daily living, social participation, and occupational function.

For these reasons, a multidimensional, consensus-based classification of visual impairment was developed that accounts for both laterality (unilateral vs. bilateral) and functional severity, as follows:

- (I) Unilateral visual impairment (visual acuity between 0.05 and 0.3 [WHO grades 1–2]; visual field 10°–20°; Other Eye: V > 0.3 and Visual Field > 20°)
- (II) Unilateral blindness (visual acuity < 0.05 [WHO grades 3–5]; visual field < 10°; Other Eye: V > 0.3 and Visual Field > 20°)
- (III) Bilateral visual impairment (visual acuity 0.05 – 0.3; visual field 10°–20°).
- (IV) Bilateral blindness (visual acuity < 0.05; visual field < 10°).
- (V) Other visual impairments (visual acuity > 0.3; visual field > 20°).
- (VI) Not classifiable (unclear, non-reproducible functional impairments; non-organic visual field deficits; developmental delays; cognitive impairments; non-organic blindness; Posttraumatic Stress Disorder; psychogenic disorders; fibromyalgia, etc.)

### Participants

In May 2023 a total of 80 rehabilitation patients (37 male, 43 female) aged between 27 and 78 years (median 58 years) were recruited. Twenty-six participants had acquired their visual impairment within the past year, 13 within the last 1–3 years, and 41 more than three years ago. The study used a convenience sample. All eligible patients admitted during the recruitment period were invited to participate; no a priori sample size calculation was performed because the study was exploratory.

**Fig. 1:**
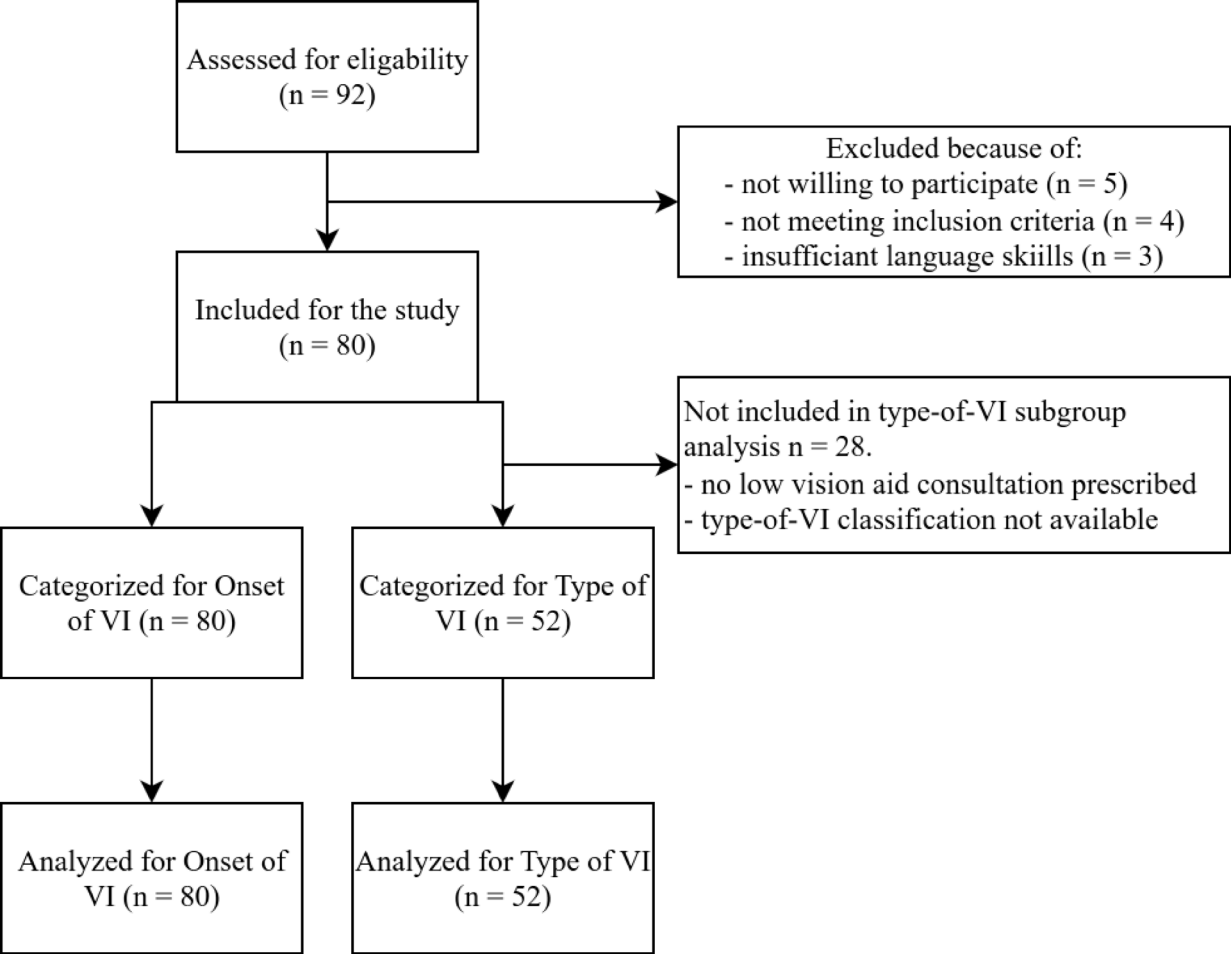
Flowchart for the Analysis Procedure.

In 52 of the 80 participants, the type of visual impairment could be determined: 10 had unilateral visual impairment, 12 unilateral blindness, 7 bilateral visual impairment, 7 bilateral blindness, and 16 had other visual disorders. For the remaining 28 participants, classification was not possible due to incomplete visual function data. Not all participants had complete ophthalmological assessments available from the department, including missing visual acuity measurements, visual field testing, or laterality information necessary for definitive classification (Fig. 1).

### Statistical analysis

After data collection, tests for homogeneity of variance and normal distribution were performed with respect to the outcome criteria, duration, and type of visual impairment. The NRS, NDI, FES-I, FABS, and MC CS criteria predominantly showed heterogeneity of variance and deviations from normal distribution. Significance testing was performed using the Kruskal-Wallis test as a non-parametric test, with effect size measures reported as f = √(η²H / (1 − η²H)); f ≈ 0.10/0.25/0.40 indicating small/medium/large effects. For better comparability with other studies, the data are reported as both mean with standard deviation and median with interquartile ranges. For pairwise post-hoc comparisons, Dunn–Bonferroni tests were used. We report adjusted p-values and effect size r (r = |z|/√n; *r* ≈ .10/0.30/0.50 indicating small/medium/large effects). The Rasch-transformed NEI VFQ-25 C logits were parametrically tested using ANOVA and reported with the effect size measure η² (> 0.01 = small effect, > 0.06 = medium effect, > 0.14 = large effect).

Regarding vision-related quality of life, Goldstein notes that the MCIDs should be considered arbitrarily as a function of distance from the zero point, as well as the value of the standard error. Goldstein refers to MCID thresholds between individuals and not to means. The MCIDs for vision-related quality of life of the subgroups to be compared were calculated using the formula: MCID = 1.96 * √ (Standard error (SE)1²/n1 + SE2²/n2), where SE1 is the mean standard error of the reference group and SE2 is the mean standard error of the subgroup being compared.

Correlations are reported with the sample size, significance level, and Pearson or Spearman correlation coefficient, depending on the data scaling. The level of significance was set at *p* ≤ .05. All analyses were performed using SPSS (version 27.0.0). Incomplete datasets were not excluded; instead, the applicable sample size for each analysis is reported.

Because of the exploratory design, no adjustment for multiple testing was applied across the overall Kruskal–Wallis tests, ANOVAs, or correlation analyses. Bonferroni correction was applied only to pairwise post-hoc comparisons following Kruskal–Wallis tests. Accordingly, statistically significant omnibus and correlation findings should be interpreted as exploratory and hypothesis-generating rather than confirmatory.

## Results

### Descriptive data

All descriptive data are presented in Table 1. Fifty-seven participants (71.3%) reported experiencing neck pain at the time of assessment. Among them, 50 (87.7%) reported chronic pain, 2 subacute pain, and 5 acute pain. These 57 individuals reported a mean score of 4.7 (SD = 1.7; Median: 4 (IQR = 3.0–6.0)) on the Numeric Pain Rating Scale and 13.5 (SD = 6.0; Median: 12.5 (IQR = 8.75–17.0) on the Neck Disability Index. Regarding motor control, 5.3% of these participants performed 0 out of 4 motor control tasks incorrectly, 12.3% performed 1/4 incorrectly, 29.8% 2/4, 40.4% 3/4, and 12.3% 4/4. Across the total sample of 80 participants, the mean FES-I score was 24.8 (SD = 7.7; Median: 22 (IQR = 19–29)), and the mean score on the Fullerton Advanced Balance Scale was 33.2 (SD = 3.6; Median = 33 (IQR = 30.25–36)). Vision-related quality of life on the NEI VFQ-25 C logit showed mean scores from 0.65 (SD = 1.91) on the Composit score, 0.67 (SD = 2.22) on the Visual Function and 0.27 (SD = 1.86) on the Social-Emotional subscale.

**Table 1:** Descriptive statistics.

	*n*	Mean	SD	Median	IQR
NP Yes	57 (71.3%)	–	–	–	–
NP No	23 (28.7%)	–	–	–	–
Duration of Symptoms		–	–	–	–
No Symptoms	23 (28.7%)	–	–	–	–
< 3 Months	5 (6.3%)	–	–	–	–
3–6 Months	2 (2.5%)	–	–	–	–
> 6 Months	50 (62.5%)	–	–	–	–
NRS	80	3.33	2.59	4.00	0.0–5.0
NP Yes	57 (71.3%)	4.57	1.71	4.00	3.0–6.0
NP No	23 (28.8%)	–	–	–	–
NDI	76	10.68	7.09	10.00	5.25–16.0
NP Yes		13.54	6.01	12.50	8.75–17.0
NP No		–	–	–	–
MC CS		–	–	–	–
0 / 4 not correct	6 (7.5%)	–	–	–	–
1 / 4 not correct	15 (18.8%)	–	–	–	–
2 / 4 not correct	19 (23.8%)	–	–	–	–
3 / 4 not correct	31 (38.8%)	–	–	–	–
4 / 4 not correct	9 (11.3%)	–	–	–	–
MC CS (NP = Yes)		–	–	–	–
0 / 4 not correct	3 (5.3%)	–	–	–	–
1 / 4 not correct	7 (12.3%)	–	–	–	–
2 / 4 not correct	17 (29.8%)	–	–	–	–
3 / 4 not correct	23 (40.4%)	–	–	–	–
4 / 4 not correct	7 (12.3%)	–	–	–	–
FES–I	80	24.81	7.68	22.00	19.0–29.0
FABS	80	33.21	3.64	33.00	30.25–36.0
NEI VFQ–25 C	69	0.65	1.91	0.67	–1.1–2.1
NEI VFQ–VF	69	0.67	2.22	0.75	–1.4–2.4
NEI VFQ–SE	69	0.27	1.86	0.71	–1.2–1.7

(Means, standard deviation (SD), Median & interquartil range (IQR) of neck pain (NP) and neck disability, fear of falling, balance ability and vision related quality of life); NRS = Numeric Rating Scale; NDI = Neck Disability Index; MC CS = Motor Control Cervical Spine; FES-I = Falls Efficacy Scale International; FABS = Fullerton Advanced Balance Scale, NEI VFQ-25 C = Composit Score, NEI VFQ-VF = Visual Function, NEI VFQ-SE = Social-emotional).

### Primary outcomes

#### Influence of the duration of visual impairment on musculoskeletal function and complaints

Significant between-group differences were observed in the Falls Efficacy Scale (FES-I), depending on the duration of visual impairment (*p* = .01; χ² = 8.94, f = 0.31). A post-hoc partial correlation analysis, controlling for age, was conducted (*r* = .269; *p* = .016) and revealed significant associations. Participants with longer-standing visual impairment reported significantly higher levels of fear of falling. Post hoc Bonferroni tests showed that only the group with onset of visual impairment < 1 year (mean = 22.65 (SD = 6.55; Median = 20 (IQR = 18–25.5)) and > 3 years (mean = 27.02 (SD = 8.05; Median = 25 (IQR = 21–31)) differed significantly, with a small effect size (*p* = .034, z = -2.53, Effect size *r* = .28) as shown in Fig. 3. The duration of 1–3 years and > 3 years narrowly missed the significance level with a small effect size of *r* = .26 (*p* = .066). No statistically significant group differences were found for the NRS, NDI, FABS, cervical motor control, or vision-related quality of life when analyzed by duration of impairment as presented in Table 2 for non-parametric and Table 4 for parametric testing.

**Table 2:** Descriptive statistics of pain intensity (NRS), neck disability (NDI), fear of falling (FES-I), balance ability (FABS), and cervical motor control (MC-CS) stratified by duration of visual impairment. Values are given as mean, standard deviation (SD), median, and interquartile range (IQR).

		N	Mean	SD	Median	IQR	p (KW)	χ² (KW)	effect size f
NRS	< 1 Year	26	3.38	2.64	4.00	0.0–5.5			
	Between 1–3 Years	13	3.31	2.81	3.00	0.0–4.0			
	> 3 Years	41	3.29	2.55	4.00	0.0–5.0			
	Total	80					0.97	0.06	0.00
NDI	< 1 Year	25	9.68	7.17	8.00	4.0–15.5			
	Between 1–3 Years	12	9.17	6.41	8.00	4.75–13.25			
	> 3 Years	39	11.79	7.22	10.00	7.0–17.0			
	Total	76					0.28	2.52	0.08
FES-I	< 1 Year	26	22.65	6.55	20.00	18.0–25.5			
	Between 1–3 Years	13	22.15	6.96	20.00	17.3–22.3			
	> 3 Years	41	27.02	8.05	25.00	21.0–31.0			
	Total	80					**0.01**	8.94	0.31
FABS	< 1 Year	26	33.42	4.11	34.00	30.5–37.0			
	Between 1–3 Years	13	33.46	3.80	34.00	31.25–37.0			
	> 3 Years	41	33.00	3.35	33.00	30.0–36.0			
	Total	80					0.78	0.50	0.00
MC CS	< 1 Year	26	2.35	1.16	3.00	3.0–4.0			
	Between 1–3 Years	13	2.46	1.13	3.00	2.0–4.0			
	> 3 Years	41	2.17	1.12	2.00	2.0–4.0			
	Total	80					0.61	0.97	0.00

p-values, χ² statistics refer to Kruskal–Wallis tests and effect size **f** comparing the three duration groups (< 1 year, > 3 years, 1–3 years).

**Fig. 2:**
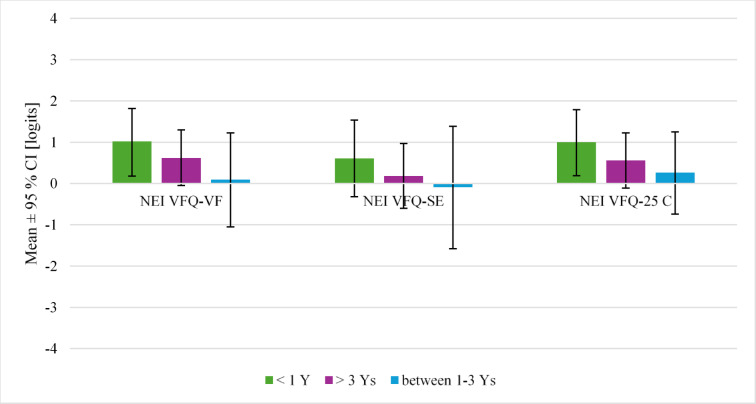
Means and 95% CIs of vision related quality of life access with NEI VFQ-VF (visual function), NEI VFQ-SE (socioemotional), NEI VFQ-25 C & onset of visual impairment. No significant differences were observed.

Regarding vision-related quality of life as a function of the duration of visual impairment, no significant differences were observed between the groups, with only very small effect sizes for NEI VFQ-25 C (*p* = .54, η² = 0.02), NEI VFQ-VF (*p* = .52, η² = 0.02), and NEI VFQ-SE (*p* = .55, η² = 0.02) as seen in Table 4 However, the calculated MCID scores as shown in Table 5 show clinically relevant differences between the subgroups. Patients whose visual impairment developed within the first year of life achieved the best results and were clinically superior to the other two groups. The lowest scores were found in the group that acquired the onset of visual impairment between one and three years (see Fig. 2). Despite the lack of statistical significance and in light of the underpowered sample size, this finding can nevertheless serve as a basis for hypothesis generation in future studies.

### Influence of type of visual impairment on musculoskeletal function and complaints

**Fig. 3:**
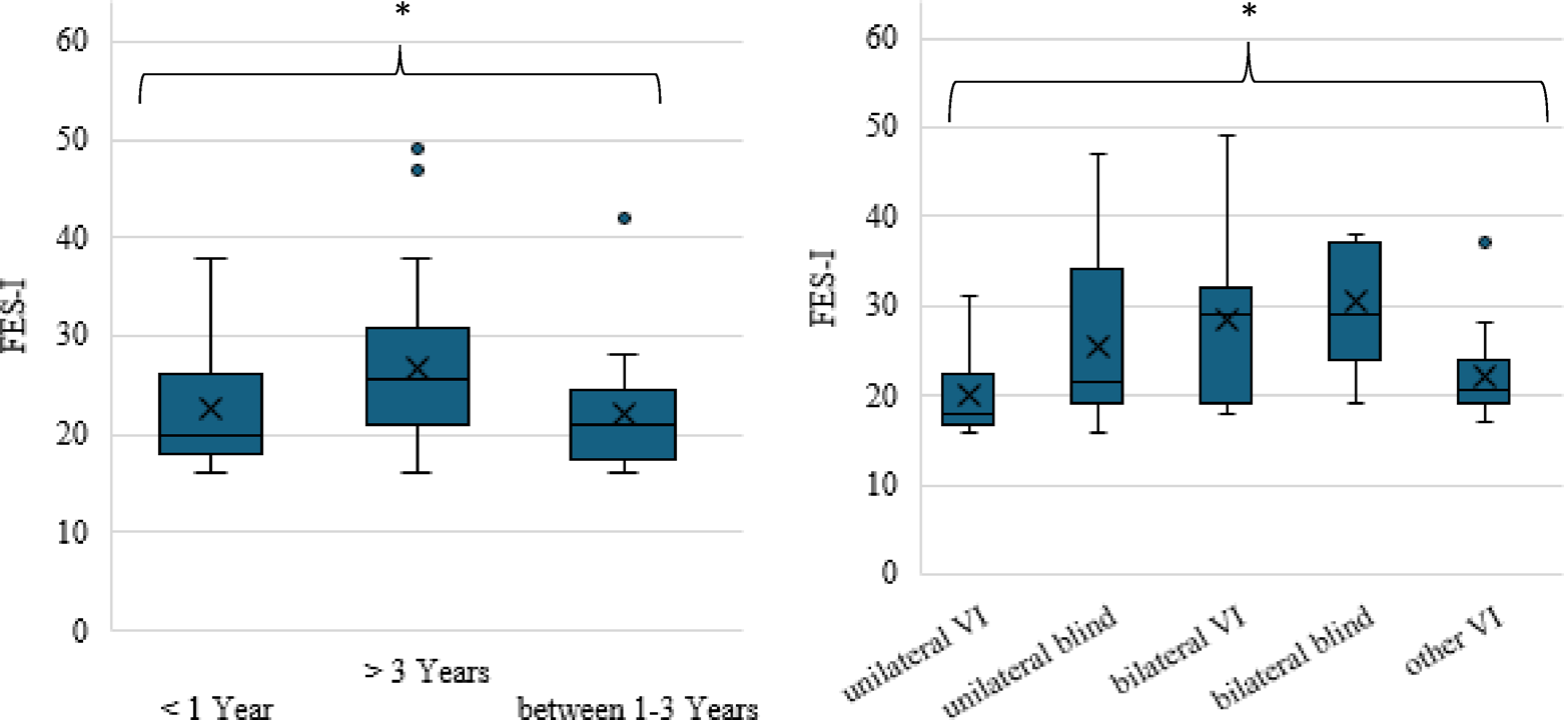
Median with IQR and mean of Falls Efficacy Scale – International (FES-I) for (A) onset of Visual Impairment (VI) and (B) type of VI; * = *p* < .05.

Significant between-group differences by type of visual impairment were observed for the Falls Efficacy Scale – International (FES-I; *p* = .03, χ² = 10.82, effect size f = 0.41). Between Group Comparisons of the different types of visual Impairment showed that only unilateral Visual Impairment and bilateral blindness passed the significance level with a high effect (*p* = .03, z = -2.97, effect size *r* = .72) as shown in Fig. 3. Although the Bonferroni-adjusted post-hoc tests did not reveal statistically significant differences between all the other subtypes of visual impairment, several pairwise comparisons showed small to large effect sizes for fear of falling. For example, patients with unilateral VI differed from those with unilateral blindness (*p* = .80, *r* = .37), bilateral VI (*p* = .33, *r* = .52) and other VI (*p* = .99, *r* = .22), indicating small-to-large effects despite non-significant p-values. Similarly, comparisons involving unilaterally blind participants showed small-to-medium effects compared to bilateral VI (*p* = .99, *r* = .14), bilaterally blind participants (*p* = .99, *r* = .35) and other VI (*p* = .99, *r* = .15). Participants with other VI showed medium effects compared to those with bilateral VI (*p* = .99, *r* = .28) and a large effect compared to bilaterally blind individuals (*p* = .24, *r* = .47).

No statistically significant group differences were found for the Numeric Rating Scale (NRS; *p* = .15, χ² = 6.83, effect size f = 0.25), the Neck Disability Index (NDI; *p* = .19, χ² = 6.15, effect size f = 0.23), the Fullerton Advanced Balance Scale (FABS; *p* = .16, χ² = 6.57, effect size f = 0.24), or cervical motor control (*p* = .43, χ² = 3.82, effect size f = 0.00), as shown in Table 3. Due to the small sample size, as well as the differing distribution within the groups, these descriptive results should be interpreted solely as hypothesis-generating.

**Table 3:** Mean, standard deviation (SD), Median and interquartile Range (IQR), p-values (Kruskal-Wallis), Chi² (χ² Kruskal Wallis) and effect size f for the outcome measures: Numeric Rating Scale (NRS), Neck Disability Index (NDI), Falls Efficacy Scale – International (FES-I), Fullerton Advanced Balance Scale (FABS), cervical motor control (MC CS) divided by Type of Visual Impairment.

	Type of VI	n	Mean	SD	Median	IQR	p (KW)	χ² (KW)	f
NRS	Unilateral VI	10	2.30	2.87	1.00	0.0–4.8			
	Unilateral blind	12	2.17	2.25	2.00	0.0–4.5			
	Bilateral VI	7	3.71	1.80	4.00	3.8–5.0			
	Bilateral blind	7	4.57	2.57	4.00	2.0–6.5			
	Other VI	16	3.81	2.43	4.00	3.0–6.0			
	Total	52					0.15	6.83	0.25
NDI	Unilateral VI	10	6.60	6.19	6.50	0.8–10.8			
	Unilateral blind	12	8.92	7.60	7.50	2.5–15.5			
	Bilateral VI	6	10.33	2.58	10.00	8.0–12.0			
	Bilateral blind	5	16.20	7.89	17.00	9.0–23.0			
	Other VI	16	11.13	7.73	10.50	5.8–15.8			
	Total	49					0.19	6.15	0.23
FES-I	Unilateral VI	10	20.10	4.65	18.00	16.8–22.5			
	Unilateral blind	12	25.58	9.72	21.50	19.3–34.3			
	Bilateral VI	7	28.29	10.73	29.00	18.8–30.5			
	Bilateral blind	7	30.43	7.30	29.00	21.5–37.5			
	Other VI	16	22.06	5.17	20.50	19.0–24.0			
	Total	52					0.03	10.82	0.41
FABS	Unilateral VI	10	34.30	3.02	34.00	31.8–36.5			
	Unilateral blind	12	32.00	3.93	31.00	28.3–35.8			
	bilateral VI	7	34.14	3.39	33.00	31.3–36.3			
	Bilateral blind	7	30.57	3.10	30.00	27.5–34.0			
	Other VI	16	33.63	3.18	34.50	30.5–36.0			
	Total	52					0.16	6.57	0.24
MC CS	Unilateral VI	10	2.70	1.25	2.50	1.8–4.0			
	Unilateral blind	12	3.50	1.24	4.00	2.3–4.0			
	Bilateral VI	7	3.14	1.07	3.00	2.8–4.3			
	Bilateral blind	7	3.71	0.756	4.00	3.0–4.0			
	Other VI	16	3.38	1.20	4.00	3.0–4.0			
	Total	52					0.43	3.82	0.00

**Fig. 4:**
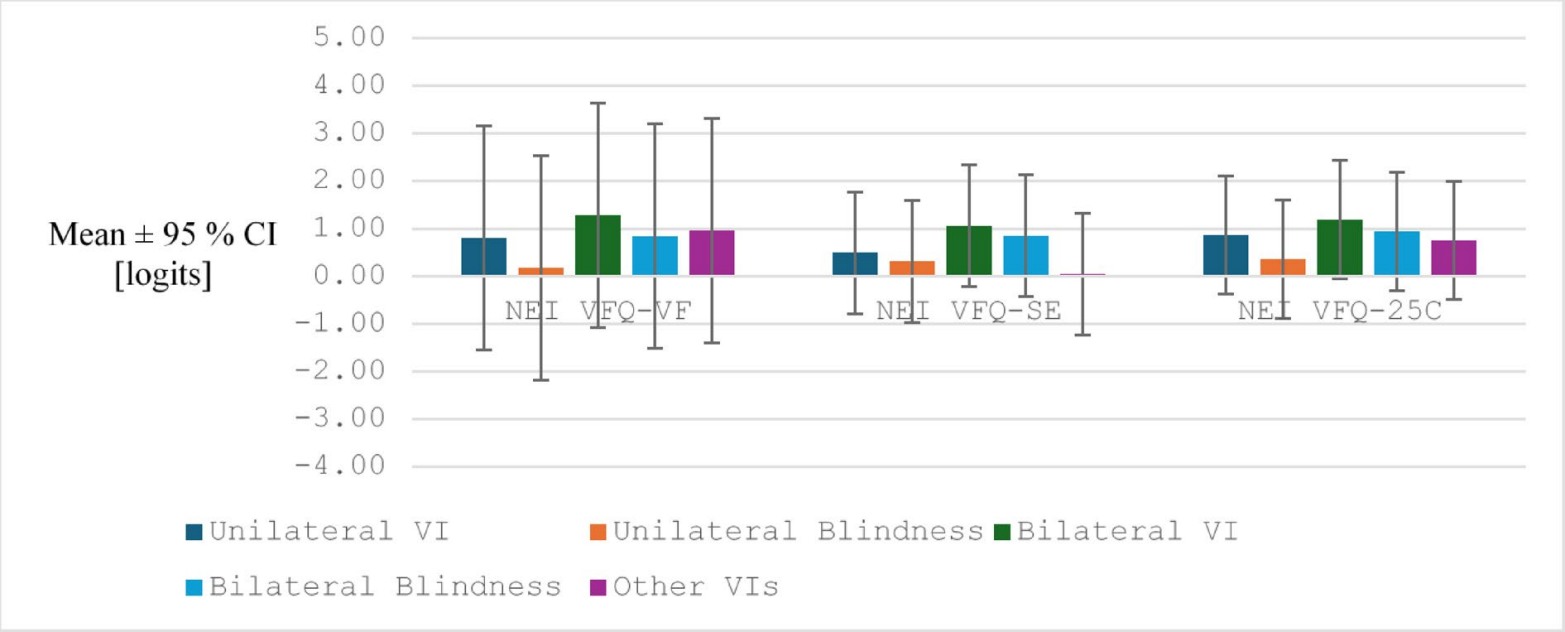
Means and 95% CIs of vision related quality of life access with NEI VFQ-VF (visual function), NEI VFQ-SE (socioemotional), NEI VFQ-25 C & on type visual impairment. No significant differences were observed.

Even with the parametrically tested outcome criteria NEI VFQ-25 C (*p* = .79, effect size η² = 0.04), as well as their subscores VF (*p* = .7, η² = 0.05) and SE (*p* = .75, η² = 0.05) no significant between-group differences were found, subdivided by type of visual impairment and mean effects of group membership on vision-related quality of life as shown in Table 6 and Figure 4.

Regarding the type of visual impairment, the MCID scores were also calculated using the formula described above and are presented in Table 7. It is noticeable that patients with unilateral blindness more frequently reported a lower vision-related quality of life than the other groups. This is particularly evident in the NEI VFQ-25 C and NEI VFQ-VF scores.

### Secondary outcome

#### Correlation of parameters

A bivariate correlation matrix is presented in Table 8. Significant correlations were found between age and reduced balance ability (r_p_ = − 0.294; *p* = .01; Pearson), as well as between female sex and higher pain intensity on the NRS (r_s_ = 0.299; *p* < .001; Spearman). The duration of visual impairment did not correlate with any of the measured parameters. However, the type of visual impairment showed a significant correlation with neck pain intensity NRS (r_s_ = 0.286; *p* = .04; Spearman), as well as with duration of Neck Pain (r_s_ = 0.324; *p* = .02; Spearman). Duration of neck pain was significantly correlated with other neck-related variables: NRS (r_s_ = 0.765; *p* ≤ .001; Spearman), NDI (r_s_ = 0.719; *p* ≤ .001; Spearman) and cervical motor control (r_s_ = 0.234; *p* = .04; Spearman). Pain intensity showed strong associations with the NDI (r_p_ = 0.724; *p* ≤ .001; Pearson), and the NDI total score was further correlated with fear of falling (r_p_ = 0.511; *p* ≤ .001; Pearson). Balance ability was significantly associated not only with age, but also with cervical motor control (r_p_ = − 0.357; *p* ≤ .001; Pearson) and fear of falling (r_p_ = − 0.29; *p* = .01; Pearson).

## Discussion

This study is the first to examine the dimensions of neck pain and balance in relation to both the duration and type of visual impairment. According to the WHO, the severity of visual impairment is currently defined by visual acuity. In this study, classification of impairment included visual acuity, the extent of visual field restriction, and whether one or both eyes were affected. This categorization was defined in advance by an interdisciplinary team of ophthalmologists, optometrists, and special educators for visual impairment. The goal was to include individuals not classified as visually impaired according to the WHO definition - such as patients who had undergone enucleation due to uveal melanoma. Classification was performed as part of the low vision aid consultation, which was not prescribed for all inpatient participants, explaining the reduced subsample size of *n* = 52, which is a major limitation of this study.

The findings highlight the relevance of neck pain and balance as clinical dimensions. In Germany, 45.7% of the general population reported experiencing neck pain at least once in the previous year^26^, compared to 71.3% reporting neck pain in this study’s sample.

The results of the FABS test further indicate that the balance performance of individuals with visual impairment does not correspond to age-appropriate levels but instead resembles that of 70- to 79-year-olds^27^, even though the median age of the patients in this study was 58 years.

When grouped by duration of visual impairment, fear of falling showed significant between-group differences, suggesting that fear of falling increases with longer-standing impairment. However, no direct correlation, but post-hoc partial correlation controlled for age was found between fear of falling and duration of impairment.

Notably, participants with recent-onset visual impairment were more likely to fall into the unilateral visual impairment group, particularly due to cases of choroidal melanoma resulting in enucleation. This indicates that the type of impairment, not just its duration, may explain between-group differences due to overlapping subgroup characteristics. No evidence could be found that prolonged visual impairment leads to adaptations in musculoskeletal conditions.

The analysis by type of visual impairment also revealed significant differences in fear of falling. A limiting factor, in addition to the small sample size, is the use of a non-validated classification of visual impairment, as mentioned above. Although some medium to large effect sizes were observed, these findings should be interpreted cautiously due to the small and unequal subgroup sizes. Descriptively, higher values for neck-related complaints and lower performance tended to occur in participants with bilateral impairment or blindness; however, these patterns were not consistent across all outcomes and should be regarded as exploratory.

No significant between-group differences were found regarding the type of visual impairment and NEI VFQ-25 C, VF und SE however, average MCID threshold as defined by Goldstein et al. exceedances were observed. The differences were primarily highlighted in the distinction between monocular and binocular involvement. Individuals with bilateral VI reported the highest scores on the NEI VFQ 25 C and its sub scores. The differences were primarily highlighted in the distinction between monocular and binocular involvement. Individuals with bilateral visual impairment (VI) reported the highest scores on the NEI VFQ-25 C and its sub scores. The lowest scores were found in patients with unilateral blindness. This frequently affects people with choroidal melanoma. The survival probability for patients with choroidal melanoma in the first year after metastasis is 50%. Overarching effects of this psychological stress on the NEI VFQ-25 C & VF scores cannot be ruled out. The substantial differences in subgroup sizes (e.g. *n* = 7 vs. *n* = 16) limit the robustness of the Kruskal–Wallis and post-hoc tests. Although the effect sizes suggest small to large between-group differences, these findings are based on small subgroups and should therefore be regarded as exploratory and hypothesis-generating. Post-hoc analysis revealed an effect size of f = 0.15, which would require a sample size of 620 individuals to demonstrate significant differences regarding the type of visual impairment based on a priori power calculations for a six-group ANOVA (α = 0.05, 1-β = 0.8).

A more severe form of visual impairment is associated with more intense and longer-lasting neck pain. This finding is not observed regarding motor control, which may indicate that factors other than motor ones, such as psychosocial or reflex mechanisms (e.g., cervico-occlusive reflex connections), are more responsible for the high prevalence of neck pain in people with VI. Similarly, fear of falling was associated with cervical dysfunction, underscoring the psychosocial dimensions of illness perception.

These findings underscore the complex biopsychosocial interplay between visual impairment and musculoskeletal health outlined in the International Classification of Functioning, Disability and Health (ICF). Visual impairment does not occur in isolation but affects multiple body functions (cervical spine, balance), activities (mobility, postural control), and participation domains (quality of life, social functioning). The documented associations between declining visual function and musculoskeletal complaints demonstrate that visual rehabilitation cannot be limited to ophthalmological interventions alone. Rather, a multiprofessional rehabilitation approach, as required by German social law (SGB IX) is essential to address the full spectrum of functional limitations experienced by individuals with visual impairment.

### Limitations

Several limitations must be considered when interpreting these results.

1. Cross-sectional study design: The design employed here collects and describes the frequencies of variables within specific categories. Consequently, any temporal or causal interpretation would not be valid.
2. Convenience sample: Due to the lack of criteria for *a priori* sample size calculation, the study is characterized by, firstly, a small overall sample size and, secondly, unequal distributions across the groups.
3. Selection bias: The correlations presented in Table 8 reveal potential confounding factors—such as the relationship between balance and age, or between neck pain severity and gender. Furthermore, the etiology of the visual impairment, psychosocial consequences, or other comorbidities may also influence pain perception or balance capabilities in this context. These factors require greater consideration in future studies.
4. The exact onset of the visual impairment and the neck pain was not recorded. Thus, while a correlation between the type of visual impairment and the duration of neck pain can be demonstrated, causal or more direct relationships remain unclear.
5. Consensus-based classification of visual impairment: The classification of the type of visual impairment was established via consensus by a panel of experts, but was not formally validated.
6. Even the examiner was unaware of Type and Onset of VI, a formal masking of the examiner was not feasible because the type and severity of visual impairment was often visually apparent (e.g., enucleation, strabismus, nystagmus, guide dog use).
7. Potential confounding factors exist among the type of visual impairment, its duration, and the specific diagnosis. The actual disease burden does not necessarily correlate with increasing severity of visual impairment or longer duration. For instance, while unilateral visual impairments are associated with choroidal melanomas, they may entail potentially greater limitations in quality of life, psychological distress, and cancer-related symptoms.

## Conclusion

Individuals with visual impairment are more frequently affected by balance and neck-related complaints. Fear of falling increases with prolonged symptoms, as well as with increasing severity of the visual impairment.

No significant correlations were found between any of the key variables and the duration of visual impairment, suggesting that these comorbidities may develop independently of the chronicity of visual impairment.

Further research with larger sample sizes is needed to confirm these findings, investigate potential causal pathways between visual impairment and musculoskeletal disorders, and evaluate the effectiveness of integrated multiprofessional rehabilitation interventions in this population.

Effective rehabilitation for visually impaired individuals should integrate allied health professionals to address balance and neck-related complaints. This addition to the multiprofessional approach aligns with the principles of the UN-CRPD, which emphasizes the removal of participation barriers through comprehensive, individualized support.

**Table 4:** Mean, standard deviation (± 1SD) and 95% Cis for NEI VFQ-25 Rasch-transformed scores in logits (composite score, visual functioning [VF], socio-emotional [SE]) by duration of visual impairment. The rightmost columns display the overall p-value from one-way ANOVA and η² as an effect-size estimate.

	Onset of VI	N	Mean	SD	95% CI		p (ANOVA)	η²
					l-CI	u-CI		
NEI VFQ-25 C	< 1 Year	22	1.00	1.87	0.16	1.8		
	> 3 Years	36	0.56	2.00	− 0.11	1.24		
	Between 1–3 Years	11	0.26	1.70	− 0.88	1.4		
	Total	69	0.65	1.91			0.54	0.02
NEI VFQ-VF	< 1 Year	22	1.02	2.10	0.09	1.95		
	> 3 Years	36	0.62	2.32	− 0.16	1.41		
	Between 1–3 Years	11	0.09	2.20	-1.4	1.57		
	Total	69	0.67	2.22			0.52	0.02
NEI VFQ-SE	< 1 Year	22	0.61	1.84	− 0.2	1.4		
	> 3 Years	36	0.18	1.98	− 0.49	0.85		
	Between 1–3 Years	11	− 0.09	1.48	-1.09	0.9		
	Total	69	0.27	1.86			0.55	0.02

**Table 5:** Mean Between-Group-Difference and calculated MCID thresholds of NEI VFQ-25 Rasch-transformed logit scores (composite score, visual functioning [VF], socio-emotional [SE]) depending on onset of visual impairment.

	NEI VFQ-25 C			NEI VFQ-VF			NEI VFQ- SE		
DIFF	1. – 2.	1.–3.	2.–3.	1.–2.	1.–3.	2.–3.	1.–2.	1.–3.	2.–3.
	0.44*	0.74*	0.30*	0.40*	0.93*	0.53*	0.43*	0.70*	0.27
MCID	0.19	0.25	0.23	0.23	0.31	0.29	0.35	0.46	0.44

1. = Onset of VI < 1 Year; 2 = Onset of VI > 3 Years; 3 = Onset of VI between 1–3 Years. * = |Diff| > MCID.

**Table 6:** Mean, standard deviation (SD), statistics and effect size of NEI VFQ-25 Rasch-transformed logit scores (composite score, visual functioning [VF], socio-emotional [SE]) depending on type of visual impairment.

	Type of VI	n	Mean	SD	Sig (ANOVA)	η²
NEI VFQ-25 C	Unilateral VI	8	0.864	1.71		
	Unilateral blind	10	0.357	1.97		
	Bilateral VI	5	1.19	1.37		
	Bilateral blind	6	0.941	1.67		
	Other VI	15	0.751	2.21		
	Total	44	0.749	1.86	0.79	0.04
NEI VFQ-VF	Unilateral VI	8	0.806	1.94		
	Unilateral blind	10	0.174	2.49		
	Bilateral VI	5	1.28	1.82		
	Bilateral blind	6	0.844	1.85		
	Other VI	15	0.959	2.59		
	Total	44	0.760	2.23	0.70	0.05
NEI VFQ-SE	Unilateral VI	8	0.488	1.73		
	Unilateral blind	10	0.310	1.75		
	Bilateral VI	5	1.06	1.18		
	Bilateral blind	6	0.851	1.52		
	Other VI	15	0.041	2.14		
	Total	44	0.407	1.77	0.75	0.05

**Table 7:** Mean Between-Group-Difference and calculated Minimal Clinically Important Difference (MCID) thresholds of NEI VFQ-25 Rasch-transformed logit scores (composite score, visual functioning [VF], socio-emotional [SE]) depending on type of visual impairment. * = |Diff| > MCID.

	Nr.	Type of VI	Difference		MCID Threshold
NEI VFQ-25 C	1	Unilateral VI	1.-2.	0.51*	0.29
	2	Unilateral blind	1.-3.	− 0.33	0.34
	3	Bilateral VI	1.-4.	− 0.08	0.36
	4	Bilateral blind	1.-5.	0.11	0.53
	5	Other VI	2.-3.	− 0.83*	0.32
			2.-4.	− 0.58*	0.34
			2.-5.	− 0.39*	0.26
			3.-4.	0.25	0.38
			3.-5.	0.44*	0.31
			4.-5-	0.19	0.33
NEI VFQ-VF	1	Unilateral VI	1.-2.	0.632*	0.36
	2	Unilateral blind	1.-3.	− 0.47*	0.42
	3	Bilateral VI	1.-4.	− 0.04	0.42
	4	Bilateral blind	1.-5.	− 0.15	0.36
	5	Other VI	2.-3.	-1.11*	0.40
			2.-4.	− 0.67*	0.40
			2.-5.	− 0.79*	0.32
			3.-4.	0.44	0.45
			3.-5.	0.32	0.39
			4.-5-	− 0.12	0.39
NEI VFQ-SE	1	Unilateral VI	1.-2.	0.18	0.54
	2	Unilateral blind	1.-3.	− 0.57	0.62
	3	Bilateral VI	1.-4.	− 0.36	0.64
	4	Bilateral blind	1.-5.	0.45	0.53
	5	Other VI	2.-3.	− 0.75*	0.58
			2.-4.	− 0.54	0.61
			2.-5.	0.27	0.49
			3.-4.	0.21	0.68
			3.-5.	1.02*	0.57
			4.-5-	0.81*	0.60

**Table 8:** Correlation Matrix of the Measured Variables Age, Sex, Onset VI (OVI), Type of VI (ToVI), Duration of Neck Pain (DNP), NEI VFQ-25 C *(25 C)*, NEI VFQ Visual Function (VF) & NEI VFQ Sozio-Emotional (SE), Numeric Rating Scale (NRS), Neck Disability Index (NDI), Motor Control Cervical Spine (MC CS), Falls Efficiacy Scale – International (FES-I) and Fullerton Advanced Balance Scale (FABS).

		Age	Sex	OVI	ToVI	DNP	SE	VF	25 C	NRS	NDI	MC CS	FES-I
Sex	Corr. Coef.	rp = 0.18											
	Sig.	0.113											
	N	80											
OVI	Corr. Coef.	rs = 0.07	rs = 0.13										
	Sig.	0.562	0.261										
	N	80	80										
ToVI	Corr. Coef.	rs = − 0.17	rs = 0.16	rs = − 0.16									
	Sig.	0.227	0.251	0.263									
	N	52	52	52									
DNP NP	Corr. Coef.	rs = − 0.08	rs = 0.16	rs = − 0.00	rs = 0.324*								
	Sig.	0.49	0.15	0.99	0.02								
	N	80	80	80	52								
SE LogIT SE	Corr. Coef.	rp = 0.04	rs = 0.11	rs = − 0.18	rs = − 0.01	rs = − 0.03							
	Sig.	0.76	0.36	0.14	0.94	0.79							
	N	69	69	69	44	69							
VF	Corr. Coef.	rp = − 0.083	rs = 0.04	rs = − 0.14	rs = 0.06	rs = − 0.00	rp = 0.821**						
	Sig.	0.50	0.76	0.24	0.70	0.99	< 0.001						
	N	69	69	69	44	69	69						
25 C LogIT CS	Corr. Coef.	rp = − 0.04	rs = 0.04	rs = − 0.14	rs = 0.03	rs = − 0.01	rp = 0.903**	rp = 0.983**					
	Sig.	0.75	0.74	0.26	0.85	0.92	0.00	< 0.001					
	N	69	69	69	44	69	69	69					
NRS	Corr. Coef.	rp = 0.01	rs = 0.299**	rs = − 0.03	rs = 0.286*	rs = 0.765**	rp = 0.06	rp = 0.06	rp = 0.07				
	Sig.	0.90	< 0.001	0.81	0.04	< 0.001	0.62	0.61	0.58				
	N	80	80	80	52	80	69	69	69				
NDI	Corr. Coef.	rp = − 0.02	rs = 0.21	rs = 0.04	rs = 0.25	rs = 0.719**	rp = − 0.07	rp = − 0.08	rp = − 0.08	rp = 0.725**			
	Sig.	0.83	0.06	0.72	0.08	< 0.001	0.57	0.50	0.50	< 0.001			
	N	76	76	76	49	76	65	65	65	76			
MC CS	Corr. Coef.	rp = 0.18	rs = − 0.05	rs = − 0.01	rs = 0.15	rs = 0.234*	rp = 0.23	rp = 0.14	rp = 0.18	rp = 0.243*	rp = 0.22		
	Sig.	0.11	0.69	0.95	0.30	0.04	0.06	0.24	0.15	0.03	0.05		
	N	80	80	80	52	80	69	69	69	80	76		
FES-I	Corr. Coef.	rp = − 0.00	rs = 0.309**	rs = 0.08	rs = 0.13	rs = 0.19	rp = − 0.06	rp = − 0.12	rp = − 0.12	rp = 0.14	rp = 0.511**	rp = 0.18	
	Sig.	1.00	0.005	0.50	0.34	0.09	0.64	0.32	0.32	0.21	< 0.001	0.11	
	N	80	80	80	52	80	69	69	69	80	76	80	
FABS	Corr. Coef.	rp = -294**	rs = − 0.02	rs = − 0.01	rs = − 0.02	rs = − 0.09	rp = − 0.09	rp = 0.08	rp = 0.037	rp = − 0.1	rp = − 0.04	rp = -357**	rp = -290**
	Sig.	0.01	0.83	0.92	0.90	0.44	0.48	0.51	0.76	0.39	0.76	< 0.001	0.01
	N	80	80	80	52	80	69	69	69	80	76	80	80

rp = Pearson correlation coefficient; rs = Spearman correlation coefficient). * = *p* < .05; ** = *p* < .01.

## Data Availability

Access to generated research data will be granted by request to the corresponding author.

## Appendix

See Tables 4, 5, 6, 7 and 8.

## Abbreviations

ANOVA: Analysis of variance
CI: Confidence interval
EMG: Electromyography
FABS: Fullerton advanced balance scale
FES-I: Falls efficacy scale – international
IQR: Interquartil-Range
MCID: Minimal clinical important difference
MC CS: Motor control cervical spine
NDI: Neck disability index
NEIVFQ-25C: Rasch-calibrated composite score of national eye institute visual function questionnaire
NEIVFQ-SE: Socio-emotional subscale of rasch-calibrated national eye institute visual function questionnaire
NEIVFQ-VF: Visual function subscale of rasch-calibrated national eye institute visual function questionnaire
NRS: Numeric pain ratin scale
RR: Risk ratio
SD: Standard dviation
SE: Standard error
UN-CRPD: United Nations convention on the rights of persons with disability
VI: Visual impairment
WHO: World Health Organization

## Declarations

### Generative AI and AI-assisted technologies in the writing process

During the preparation of this work the author(s) used ChatGPT 5 and Perplexity to improve English language. After using this tool/service, the author(s) reviewed and edited the content as needed and take full responsibility for the content of the publication.
